# Pathology and Practice *(Medical)*

**Published:** 1818-01

**Authors:** 


### II. PATHOLOGY and PRACTICE *(Medical)*.

1. *Gout.* Until the able treatise of Dr. Scudamore* made its appearance, there existed nothing like any fixed principle, or sound and rational practice, in the treatment of gout. One of the most painful and ungovernable diseases to which the human system is exposed, was commonly abandoned, with sullen resignation, to the tardy influence of time and flannel; or subjected, in a fit of desperation, to the coarse and dangerous prescriptions of blind empiricism. Hopelessness of success seems to have gradually engendered in the mind of the medical practitioner, indifference as to the object. The phænomena of a malady, whose depredations art daily proved itself inadequate to arrest, and often even to mitigate, were, at length, less vigilantly observed, and their nature and origin less closely investigated. To this cause may, perhaps, in great measure, be attributed the obscurity in which the pathology of gout has hitherto lain involved, and the scantiness of morbid anatomical description respecting it, which, considering the frequency of the disease, we find on record. Part of this void Dr. Scudamore has filled up in a manner, which precludes to us any hope or effort at improvement. But we shall sedulously embrace every opportunity of presenting to our readers accurate reports of the morbid appearances which the victims of gout exhibit, and of inciting them to similar researches; fully convinced that in this quarter, a fertile and interesting track yet lies unexplored.

The subject of the first important case† of this kind, which has fallen in our way, was a man aged sixty-three. He had been gouty for many years, and had annually suffered four or five very violent paroxysms, with calcareous depositions in the joints. On dissection, all the soft parts were found in a friable condition. An effusion of fluid, of a sensibly acid smell, had taken place between the abdominal muscles and peritonæum, as well as into the abdominal cavity. The omentum was destroyed, with the exception of some small fragments, which were destitute of fat and much discoloured. The liver displayed double its ordinary volume, and was considerably indurated. On the surface of the organ, beneath its peritonæal covering, were scattered calcareous concretions, of the size of millet seeds, with intervening patches of a dark red colour. Its internal structure contained many larger concretions of the same kind.

---

* *Treatise on the Nature and Cure of Gout*, &c. London, 1816.

† *Leichenöffnung eines im 63 sten Jahre an eingewurzelter Gicht verstorbonen Mannes; nebst einegen kurzen Bemerkungen*, Von Dr. Ideler &c. *Journal der Practischen Heilkunde*, Nov. 1815.

The spleen also, of a natural size but much softened, presented, beneath its external membrane, similar substances; as did the surface of the kidnies, which, in every other respect, were, like the rest of the urinary system, perfectly natural; and the descending aorta from the vicinity of the lumbar vertebræ to its division in the pelvis. All the other abdominal organs were in a natural condition, except that the large intestines were greatly distended, and their veins enlarged and varicose, particularly those of the rectum. The cranium and thorax were not inspected.

Dr. Ideler, who is the author of this case, afterwards proposes the three following queries:—What caused the rapid increase of volume of the liver during the gouty paroxysms which occurred in the last years of the patient's life? Whence proceeded the acid smell of the effused serum? What, in this subject, became of the omentum? We shall briefly trace him in the attempt at the respective solutions of them. In the first place, Dr. Ideler observes, that in many arthritics, the disease has a tendency to act upon the liver previously to the eruption of the paroxysm; and that, in his opinion, the gastric symptoms, then developed, may be attributed to such action. Secondly, he explains the acid smell of the effused fluid by the excess of phosphoric acid in gouty subjects; who continue well so long as this excess is carried off by the skin and kidnies. Thus the acid no longer exists in these subjects, in an evident manner, when the paroxysms are developed, but manifests itself afresh after their decline: so that the approach of a fit may be predicted, when litmus paper ceases to be reddened by immersion in the urine. Lastly, he refers the destruction of the omentum to the effect of chronic inflammation, terminating in suppuration and absorption of the pus.

2. *Intestinal Affections.*—The generation of flatus in the intestinal canal is a phænomenon, the study of which, whether viewed in its physiological or pathological relations, becomes alike important. And we think that it has yet by no means attracted the degree of attention to which, either as a symptom of disordered function in the chylo-poietic system, or as frequently becoming, itself, a source of other formidable and distressing symptoms, it is entitled. Hence, we have read, with peculiar interest and satisfaction, some "Observations upon Borborygmi considered as symptomatic of certain abdominal affections,"† by a French physician. Our analysis of this valuable memoir we may be allowed to preface by observing that intestinal flatus varies considerably as to its source, its chemical constitution and properties, and the situation occupied by it. In the first place, it may result from air introduced into the

---

† *Réflexions sur la séméiotique et Observations sur les borborygmes, considérés comme signes de quelques affections du bas ventre. Bulletin de l'Athén. de Méd. de Paris*, Juin, 1817. The former part of this memoir seems to contain nothing worthy of particular notice. Edit.

stomach with the alimentary substances, or from the gaseous fluids extricated by them in the different changes of which they are susceptible.* Secondly, it may consist simply of carbonic acid, completely inodorous, or of carburetted or sulphuretted hydrogen, with a smell varying both in character and intensity. Commonly also, it contains a portion of azote, admitted into the stomach in the interstices of the alimentary bole. Lastly, it may be extricated in the stomach, or in the small or large intestines. The absorption of the carbonic acid of intestinal flatus by injection of lime-water into the rectum, and the condensation and consequent diminution of the volume of the gas by topical application of ice to the abdomen, are the principal indications by which the practice of some of the continental physicians, in these cases, appears to be directed.† Dietetic restrictions constitute, according to our experience, the most effectual means of remedying that disordered condition of the stomach and bowels upon which the *morbid* generation of the intestinal gases is most commonly dependent.‡

After noticing the false ideas which the older writers, destitute of the light of pneumatic chemistry, entertained upon the nature and origin of flatulence; and the error into which many have fallen in regarding it, and arranging it in their systems of nosology, as an essential disease, M. Moreau, the author of the memoir, proceeds to claim the attention of practitioners to that variety of flatus termed, from the noise produced by it in traversing a certain portion of the intestinal canal, *borborygmus.* Viewing the subject merely in its relations with symptomatology, he asserts, from many facts hereafter to be detailed, that, whenever borborygmi cease completely to be heard at any particular point of the intestinal tube, an obstacle of some kind, opposing their passage, exists at such place. Hence, he thinks himself justified in regarding this phænomenon as a sign of some lesion of the alimentary canal obstructing the issue of the intestinal gases; and infers that it may become an useful indication in the diagnosis and treatment of abdominal diseases. From the commencement of his practice, Moreau had remarked this symptom; but, the opportunity of correct investigation rarely presenting itself, he remained long undecided respecting it. At length, cases occurred which completely dissipated his uncertainty, and induced him to consider the observation as a fact interesting to pathology. These cases are five in number. We shall cursorily trace their more prominent features and result.

---

* The presence of the intestinal gases is supposed to be, in some cases, the result of a peculiar action of the exhaling vessels. The experiments which have been hitherto brought forward in support of this opinion are, however, inconclusive. A thesis in favour of it was published by Gerardin, at Paris, in 1814.

† *Dictionaire des Sciences Médicales,* tome iii. Art. *Borborygme.*

‡ For an account of the nature of the intestinal gases of the human subject in a state of health, see *Med. Chir. Journ.* vol. iii. p. 159.

A woman, aged 50, experienced much difficulty in voiding her fæces, which were hard and elongated like a wire. This circumstance arose from contraction of the rectum, consequent on the repeated inflammations of which that organ had been the seat. Notwithstanding the habitual use of injections, the evacuation became progressively more difficult: and, in the hope of obtaining relief, the woman ate a large quantity of cherries, and swallowed the stones. Soon after this, the discharge of fæces was completely suppressed. Colic, and tumefaction of the abdomen ensued. Borborygmi were heard at the left inferior part of the inguinal region, where they seemed to terminate. After having investigated the history, and ascertained the actual state of the case, M. Moreau was led to suspect that an accumulation of cherry stones impacted in the narrowest part of the rectum, formed an obstacle to the escape of flatulence and fæces. Injections, and the introduction of an elastic gum catheter, were tried in vain. The colic increased in violence, accompanied with borborygmi, which, descending from above, terminated at the point before mentioned. Vomiting and excessive enlargement of the abdomen supervened. The colon, enormously distended, might be traced beneath the abdominal parietes. No stool was voided. Oppression, coldness of the extremities, and failure of the pulse speedily followed. The countenance was entirely changed. The case was now desperate; but, in expectation of affording some relief, a trochar was plunged into the descending colon. A quantity of gas escaped from the orifice; the abdomen sank, and the woman felt better; but the intestines were soon distended afresh, and death speedily ensued.—On dissection, the intestines were found excessively dilated. The mucous membrane of the rectum presented, at its centre, a thickening which occupied its circumference for about two inches. The parietes of the intestine were, in this place, changed into a cartilaginous substance. The thickening of the mucous membrane had so far diminished the diameter of the rectum, that it would scarcely admit the little finger. Above this contraction were found seven cherry stones, by which the intestinal tube was completely obstructed.

A man had been, for several days, without stools. The injections employed, did not pass the rectum, and were soon rejected. He had colics and a distended abdomen. Borborygmi were heard; and the noise seemed to cease in the left iliac region, just above the groin, without any escape of flatus per anum. The patient complained of a dull deep-seated pain in the hypogastrium. M. Moreau suspected, on examination, the existence of some obstruction to the passage of the fæces in the lower extremity of the descending colon. Hence diluents, laxatives, injections, the warm bath, and a rigid diet, were prescribed. The introduction of a sound into the rectum threw no light on the nature of the case. Vomiting and inclination to go to stool, and great abdominal distension, ensued. After some days, pus was voided by the rectum; and, from that time, the vomiting and tension of the belly disappeared, the course of the fæces was restored, and the patient thought himself well. Abandoning his dietetic restrictions, he now indulged the cravings of his appetite. Some days afterwards, pains were felt in the abdomen. The patient became pale, his extremities cold, his pulse small and feeble, and his body covered with a clammy sweat. He expired suddenly.—On opening the abdominal cavity, twenty-four hours after death, there escaped a great quantity of fæces, which had been effused into it. The inferior part of the descending colon displayed, near the origin of the rectum, an orifice with livid, and, as it were, scolloped borders, establishing a communication between the colon and abdominal cavity. The state of the parts surrounding the aperture is not mentioned.

Individuals, affected with retention of urine, symptomatic of general disease, were the subjects of the three last cases, in which the compression, exercised by the (distended) bladder, obstructed the escape of fæces and intestinal flatus: and in these, as in the preceding instances, the author asserts, that the noise of the borborygmi in the abdomen ceased precisely at the point of the hypogastric region, where the urinary organ pressed on the intestinal canal. He adds, that, after the evacuation of the former by the catheter, a great quantity of flatus and fæcal matter was discharged from the rectum. From these facts, and others previously observed by him, M. Moreau deduces the following corollary: "In all cases, where an obstacle to the passage of excrement and flatus exists in the large intestines, the borborygmi, which are heard in the abdomen, take a course from above downward; and the point where they terminate, indicates precisely the seat of the obstruction." So certain does the author consider the results of his experience on this subject, that he hesitates not to erect upon them a new aphorism: *Quo silet borborygmus, ibi quodcumque obstaculum.* Future observation can alone determine the correctness of this medical axiom. However it be, the cases contained in Moreau's Memoir, and particularly the two first, display considerable interest, and forcibly bring to our recollection the "Cases in elucidation of the theory of Tympanitis," about a year since published by M. Bricheteau.* Of these we propose shortly to render an account.

*Melæna*, the morbus niger of some writers, is a disease of rare occurrence, and very few descriptions of the internal morbid appearances which it displays are to be found on record. M. de Lens,† a French practitioner, reports, that he has seen a man die, in a few days after having voided pure blood by the mouth, and a black and pitchy matter by the rectum. The case had been treated as hæmoptysis. On dissection, the pulmonary organs were sound, and the transverse colon filled with a substance precisely like that which, during life, had been evacuated from the bowels.

* *Bulletin de l'Athén. de Méd. de Paris*, Septembre 1816.

† *Bulletin de l'Athén. de Médic. de Paris*, Mai 1817.

PATHOLOGY and PRACTICE *(Surgical).*

1. *Tumour of the Tongue successfully treated.* The following case of "cure of enlargement of the tongue"* is too striking, and bears too closely upon a recently proposed method in the treatment of cancer, to require introductory comment or remark. A woman, aged 24, applied to M. Fréteau, of Nantes, with considerable enlargement of the tongue, which appeared cancerous, and projected four inches from the mouth. An offensive sanies flowed constantly with the saliva from the whole cavity; and the exhausted patient could take only broth and milk, by means of a reed. She had been in this state six weeks. Leeches, scarifications, and blisters, had been employed in vain; and amputation was proposed as the last resource. M. Fréteau, however, resolved to try the effect of pressure. After removing the four inferior incisor teeth, by which the tongue had been much cut and irritated, he encircled the protruded portion of the organ, as near as possible to the mouth, with some turns of flat elastic silk, and secured each turn with the point of a needle. Thus the whole was covered, and felt like a compressed sponge. It was evidently diminished in volume. Three plates of elastic gum, placed above, and on the sides of the tongue, to defend it from the action of the teeth, were fixed by fresh turns of the silk binding; and the whole sustained by a compress attached to the posterior superior part of the head. The patient found great relief from this apparatus. The bandage having become loose, was removed at the end of forty-eight hours; and the tongue, softened and reduced to half its former bulk, re-entered the mouth without difficulty; but, from an increase of breadth, it got engaged among the teeth. This inconvenience was obviated by tonic gargles, which served farther to diminish the volume of the organ. A painful induration was now discovered on its right edge. It clearly arose from the irritation of a ragged tooth; and hence the latter was extracted. On the third day from the commencement of the treatment, the tongue was wholly reduced. On the fifth, the patient could take soup without the assistance of her reed. On the eighth she laid aside the chin-cloth, by which the lower lip and jaw had been supported. On the twelfth, she could execute all the masticatory movements; and on the fifteenth, she returned home perfectly recovered.

2. *Lesions of the Stomach.* Wounds, penetrating into the cavity of the stomach, were once considered as almost necessarily fatal. This notion, however, reiterated observation has proved to be erroneous. How severe a degree of mechanical injury may be inflicted on the delicate and important organ in question, with ultimate impunity to its functions, the case which we are about to

* *Guérison d'une intumescence de la langue, avec prolongement hors de la bouche.* Par M. Fréteau, &c. *Gazette de Santé,* Mai 1817.

transcribe* is well calculated to shew.—A boy, aged 12, employed in pruning trees, fell, about two hours after dinner, August the 18th, from the height of ten feet, upon a hedge. The abdomen sustained the whole shock; and a pointed branch of hawthorn, nearly two inches in circumference, entered the stomach by the left hypochondrium. The organ presented, through the wound, an opening of an inch and a half in extent at its large extremity, and parallel to its great border. The boy was visited, about an hour after the accident, by a surgeon. His shirt was soaked with blood, and the aliment previously taken. The stomach was protruded through the wound, but not strangulated. The retroversion of the borders of the rupture externally prevented the spontaneous reduction of the viscus. The extremities were cold; the pulse small, contracted, and, at times, "convulsive;" the face bathed with sweat, and the features altered. Hiccup and vomiting were induced by the slightest movement. The patient having been conveniently placed, and the wound cleaned, a portion of the gastro-colic omentum, uninjured, came into view. The membranes, forming the hernia, were inflamed, and pressure on the epigastrium re-excited the hiccup and vomiting. The double waxed threads were now passed, by means of a curved needle, through the gastric membranes, in a direction parallel to the external wound; and the stomach, emptied by the effects of vomiting, was readily reduced. The extremities of the ligatures were retained externally to the wound of the integuments, in order to prevent effusion into the abdomen; while an assistant made the necessary sutures, which were fastened in such a manner, that they might be tightened, or relaxed, at pleasure. The wound was then covered with *charpie*, and compresses soaked in a mucilaginous decoction: and the whole supported by a body-bandage. A most rigid diet was prescribed.—19th. Fever and tension of the pulse came on, with severe peritonitis for about twelve hours, although venæsection had been practised.—20th. Debility, with continual sleeplessness. Tension and pain of the abdomen, relieved by the operation of a glyster.—23d. Slight fœtid suppuration; no fever.—24th and 25th. Part of the fluids, which had been drunk, issued from the wound.—26th. General uneasiness; periodical colic at midnight for four successive days, with lancinating pains in the wound.—31st. One of the ligatures detached, and abundant suppuration, bringing with it some sphacelated portions of the gastric membranes.—Sept. 2d. All the ligatures favourably detached; separation of the gangrenous portions continuing. The wound contracting by adhesion of the membranes

* *Observation sur une plaie de l'Estomac avec hernie, produite par un corps contondant, et Réflexions de M. le Prof. Percy. Bulletin de la Faculté de Médecine de Paris,* &c. 1817, No. V. We think, with the Professor, that the term *piquant* would be very properly substituted for that of *contondant,* employed in this title. EDIT.

of the stomach to the abdominal parietes. Insatiable hunger.—9th. Swelling of the umbilical region; a tumour observed in the left groin, which suppurated and was opened on the 29th.—Oct. 4th. This opening closed after the evacuation of a large quantity of fœtid pus. No change exhibited by the integuments.—30th, Sudden œdema of the left side of the thorax and abdomen.—31st, Swelling of the face, with orthopnœa. An injection of cinchona, and general frictions with camphorated spirit, prescribed thrice a day. Detachment of the epidermis, previously dried by the emaciation of the subject, soon took place, followed by cutaneous perspiration; the urine became abundant; and the boy regained his ordinary bulk and health. He has acquired the habit of lying upon his back, when in pain, to take his food; and, on rising, he resumes his labour. In order to assure himself of the condition of the injured parts, the surgeon passed his finger round the protruded portion, and into the aperture, of the stomach; and his hand was instantly filled with part of the fluids taken since the accident, and simultaneously ejected by the œsophagus and the wound. In this uncommon case, the stomach, when pressed between the fingers, exhibited an alternate contraction and relaxation.

We may here not inappropriately mention the singular case * of a melancholic, stated at one of the French medical societies, by Royer-Collard. The insanity of this man consisted in his thinking that there existed a determined conspiracy against his life, under the direction of a powerful triumvirate. Upon every other subject he reasoned correctly. He was a man of talent and literary acquirements, and expressed his ideas with remarkable elegance and purity. He had thrice been the inmate of a lunatic asylum. Upon complaining, that, about three years previously, he had, in a fit of despondency, swallowed an iron fork, he was subjected to repeated examinations, without, however, the presence of any foreign body being detected. His digestion appeared to be good, and the colour of his fæces natural; yet, in this respect, he attempted to practise imposition. He at last hung himself with a bandage, which had been employed to fasten on a blister. On dissection, the *stomach*, otherwise healthy, was found to contain a fork of japanned iron, the prongs of which were closely pressed together: all the other viscera were sound. This case, and that of the knife-eater, whose body was, some years since, examined at Guy's hospital, to which it bears considerable analogy, strikingly illustrate the proposition with which we set out, respecting the occasional insensibility of the stomach to very severe degrees of mechanical injury and irritation. They, moreover, inculcate the propriety of patient and minute attention to the statements of the sick, however strange or extravagant they may at first sight appear.

3. *Operations on the Bladder.* The importance of attention to

* *Bulletin de l'Athén. de Méd. de Paris,* Nov. 1816.

two circumstances, in the introduction of instruments into the bladder, cannot be too strongly impressed upon the recollection of the young surgeon. These are the employment of a female catheter, furnished with a proper guard to prevent the possibility of the complete escape of the instrument into the urinary organ; which may otherwise take place, from a sudden spasmodic effort of the urethral muscles; and the selection of good bougies and catheters, especially in operations on the male subject. From a culpable neglect of these precautions, many a practitioner has doomed his patient to years of dreadful, and perhaps hopeless, suffering, and brought down irreparable disgrace upon his own head. The fact, cursorily mentioned below,* will sufficiently exemplify the unhappy consequences of inattention to the first of these points; and those, resulting from neglect of the latter, will be exhibited by the case † which has given rise to these remarks. A French surgeon, in performing the operation of *lithotomy upon a male*, found the stone, which was very brittle, one inch and a half long, and eight or nine lines thick, traversed, in the direction of its greater diameter, by a piece of elastic gum catheter. The extremities of it were yet free from incrustation. This extraneous body had, doubtless, accelerated, if not wholly given rise to, the formation of stone, by irritating the bladder, and acting as a nucleus for the deposition of calcareous matter. M. Picod, the operator, asks whether, in the event of an instrument breaking in the bladder, it would be better to effect a gradual dilatation of the urethra, and prevent the incrustation of the detained fragment by occasionally striking it with a silver catheter, until a natural presentation in the canal may render its removal practicable; or awaiting its decomposition by the urine, and consequent spontaneous expulsion, piece-meal, with that fluid.‡

---

* Some years since, a surgeon, practising in the country, was required to introduce the catheter, for a lady labouring under retention of urine. One day, during the operation, he was observed to exhibit signs of confusion, and to quit his patient under considerable embarrassment. On that day he abruptly left his home, and was never seen afterwards. The lady passed several years of dreadful suffering, attributed by herself, and the professional gentleman on whom the treatment of the case devolved, to a spontaneous aggravation of the urinary complaint. At length, an abscess presented in the sacral region. The surgeon had occasion to puncture it. His instrument came in contact with some unusually hard substance imbedded in the centre of the abscess; and, with the dressing forceps, he extracted, to his utter astonishment, a blackened *female catheter*. Thenceforth, all the lady's sufferings were happily terminated; and reflection on the circumstance served to explain the abrupt and mysterious disappearance of her former medical attendant. The writer of this had, in his younger years, narrowly escaped a similar accident. Edit.

† *Observation sur le danger d'employer de mauvaises sondes de gomme elastique; par A. Nicod,* &c. — *Journal de Médecine, par Leroux,* Oct. 1816.

‡ A French writer, noticing this case in another Journal, says, that he

Pathology and Practice *(Obstetrical)*. 1. *Gastrotomy.* This operation, hitherto almost invariably fatal in its termination, was successfully performed at Parma, on the 9th of August last, by Professor Cecconi.* The subject of it had suffered *rupture of the uterus,* during the process of parturition, about the middle of the preceding night. The fœtus, with its membranes, had escaped into the abdomen. An accoucheur, summoned about six o'clock in the morning, ascertained the nature of the case, and proposed the operation, as the only mean of rescuing the patient from destruction. Two minutes sufficed to make the incision and extract the fœtus, which weighed *about* fourteen pounds, and appeared to have been some hours dead, from the abdomen. The whole operation was terminated in eight minutes, and the woman had a favourable recovery. A more particular account of this rare and interesting case will be shortly published.

2. *Labour retarded from uncommon Causes.* Nothing, perhaps, among the ordinary occurrences of midwifery practice, more strongly excites the alarm of the patient and her attendants, or is more calculated to disconcert the young accoucheur, and rob him of that coolness and self-command which it is of the utmost importance to his character and success, that he should never for a moment lose, than an unexpected obstacle to the complete extrication of the fœtus, when already in part expelled from the organs of the mother. Hence, it behoves the practitioner to make himself familiarly acquainted with every variety of impediment to parturition which has yet been observed, and so to exercise his mind, in the calm season of retirement, upon a review of the obstructing causes, that, in the hour of trial and distraction, the whole may, with facility, be recalled. From such considerations, we are induced to record the case † of a woman, aged 32, who, at the close of her fifth pregnancy, was seized with pains of extraordinary violence, duration, and frequency. The uterine orifice was much dilated, and the membranes duly distended with the waters during each pain. The head appeared to present naturally ; the woman was healthy and robust. On an increase of the pain, the membranes at length broke; and the head and superior part of the trunk were immediately disengaged by the attendant midwife, but the inferior portion was detained by some insuperable obstacle, and a surgeon consequently summoned. He at first tried, but in vain, to turn the body of the child, in order to facilitate its passage through the inhas known an instance wherein the broken portion of a catheter was fortunately extracted from the urethral canal by a cautious re-introduction of the instrument from which it had been separated. *Bibliothèque Médicale,* Janvier 1817.

* *Exemple heureux d'une operation de Gastrotomie. Gazette de Santé,* Nov. 1817. (Taken from the *Gazette of Parma,* Sept 20th 1817.)

† *Accouchement rendu laborieux par une tumeur existant chez le fœtus.—Recueil périodique de la Société de Médecine de Paris,* Juin 1817.

ferior apperture of the pelvis. He then introduced his right hand, well oiled, along the abdomen, to ascertain the cause of obstruction; and felt an enormous tumour, arising from the sub-pubic region of the fœtus, and forming a kind of wedge between its thighs. The tumour was hard, tense, and elastic, like the distended membranes in a strong labour-pain. It burst on pressure, and gave issue to a fluid, resembling in appearance, and equalling in quantity, the liquor amnii. The extraction was then terminated without difficulty, and the placenta came naturally away. Upon examination of the infant, which was living and a female, the tumour appeared to be formed by a prolongation of the skin of the abdomen and superior part of the thighs, and descended to the middle of the legs. It might have contained about two *pintes* of fluid. The orifice of the vulva was placed directly beneath it, just at its origin, posteriorly from the lower part of the pubes, and summit of the thighs. The infant was, in other respects, well formed, but died in a few hours. The body was not examined.

---

### PART IV.

### MEDICAL MISCELLANIES.

### THE PORTE FEUILLE, No. X.

OR,

DEPOT FOR DETACHED FACTS AND INTERESTING PHÆNOMENA, OCCURRING IN THE PRACTICE OF THE FRIENDS OF THE MEDICO-CHIRURGICAL JOURNAL.

"——— Trahit quodcunque potest
Atque addit acervo ———"

*Punctured Wound of the Eye.*

